# The Mini-CAARMS: Development and Validation of a Short Version of the Comprehensive Assessment of AT Risk Mental States to Facilitate Preventive Psychiatry

**DOI:** 10.1093/schbul/sbaf146

**Published:** 2025-09-18

**Authors:** Alberto Stefana, Dominic Oliver, Andres Estrade Vaz, Matilda Azis, Stefano Damiani, Laura Fusar-Poli, Natascia Brondino, Umberto Provenzani, Ilaria Bonoldi, Peter Uhlhaas, Paolo Fusar-Poli

**Affiliations:** 1 Department of Brain and Behavioral Sciences, University of Pavia, Pavia, Italy; 2 Center for Behavioural Sciences and Mental Health, National Institute of Health, Rome, Italy; 3 Department of Psychiatry, University of Oxford, Oxford, UK; 4 NIHR Oxford Health Biomedical Research Centre, Oxford, UK; 5 OPEN Early Detection Service, Oxford Health NHS Foundation Trust, Oxford, UK; 6 Early Psychosis: Interventions and Clinical-Detection (EPIC) Lab, Department of Psychosis, King’s College London, London, UK; 7 Department of Child and Adolescent Psychiatry, Charité Universitätsmedizin, Berlin; 8 NIHR Maudsley Biomedical Research Centre, London, UK; 9 OASIS Service, South London and Maudsley NHS Foundation Trust, London, UK

**Keywords:** psychosis risk, psychometric assessment, semistructured interview, ARMS, CHR, schizophrenia, prevention, CAARMS

## Abstract

**Background and Hypothesis:**

Accurate detection of individuals at risk for psychosis with established psychometric instruments such as the Comprehensive Assessment of At-Risk Mental States (CAARMS/2006) is crucial to implement effective preventive strategies. However, the CAARMS/2006’s scalability is limited by its lengthy administration. This study developed and validated a shorter CAARMS/2006 version: the mini-CAARMS.

**Study Design:**

A total of 490 participants (mean age 23.8±5 years) underwent the full CAARMS/2006 interview (60 items, all individually scored). The development and validation of the mini-CAARMS employed advanced statistical methods: regularized logistic regression with a LASSO penalty within a nested cross-validation framework to retain core items; Cohen’s κ, Harrell’s C index, sensitivity, specificity, balanced accuracy, positive (PPV) and negative (NPV) predictive values to assess performance; Monte Carlo simulations to assess the 2-year prognostic accuracy for predicting psychosis onset. A sensitivity analysis developed and validated an ultra-mini-CAARMS.

**Study Results:**

The mini-CAARMS retained only 23 items and demonstrated excellent accuracy compared to the full version: Cohen’s κ 0.90; Harrell’s C index 0.93, sensitivity 95.6%, specificity 100%, balanced accuracy 97.8%, PPV 100%, NPV 86.3%. The prognostic sensitivity and specificity of the mini-CAARMS were 82.2% and 55.1%, respectively. Sensitivity analyses further developed and validated an ultra-mini-CAARMS (based on 12 items only), which achieved comparable performance, with κ 0.90, C index 0.91, sensitivity 94.4%, balanced accuracy 97.2%, PPV 100%, NPV 82.2%, prognostic sensitivity and specificity 81.2% 55.1%, respectively.

**Conclusions:**

The mini-CAARMS maintains high accuracy while substantially reducing administration time, and it could facilitate scalability of preventive psychiatry in low-resource settings.

## Introduction

The onset of psychotic disorders is typically during adolescence and early adulthood^1^ and they are estimated to have a mean lifetime prevalence of 9.57 per 1,000 individuals.^2^ Globally, psychotic disorders (e.g. schizophrenia) alone affects approximately 20 million people.^3^ Schizophrenia ranks among the leading causes of years lived with health-related disability,^3^ whose economic burden is up to 1.65% of the gross domestic product.^4^ Furthermore, psychosis causes significant disruptions in the lives of those affected and impose a substantial burden on their families.^5^

The onset of a psychotic disorder often limits the opportunities to alter its course.^6^ A recent meta-analysis found that 78.3 % of individuals who later developed psychosis experienced a prodromal phase before disorder onset, underscoring a key window for early detection and subsequent preventive intervention.^7^ However, with the introduction of the clinical high risk for psychosis (CHR-P) construct,^8^ preventive strategies have become viable, even in national health system, complementing and enhancing early intervention efforts focused on the first episode of the disorder.^9,10^ The CHR-P construct is defined by the presence of attenuated positive psychotic symptoms – symptoms that are not severe or persistent enough to meet the full ICD/DSM criteria for a psychotic disorder.^11–15^ Recent meta-analytic pooled estimates indicate that the prevalence of CHR-P state is 1.7% in the general population and 19.2% in clinical populations.^16^ Approximately 20% of CHR-P individuals transition to psychosis within two years, and 35% within ten years,^15^ leading to poorer functioning^17^ and a high personal burden.^5,18^ Among those who do not transition, functioning tends to improve; however, over half of the individuals do not experience full remission from their psychotic symptoms after three years^19–21^ and continue to experience persistent comorbid mental disorders.^22,23^

Effective preventive strategies for CHR-P individuals are available.^11,24,25^ Preventive psychiatry for psychotic disorder is essentially grounded on three core components: our ability to detect most individuals at risk, to prognosticate their outcomes, and offer them effective preventive interventions.^26^ Among these, accurate detection of young people at CHR-P is the first rate-limiting step. Detection of CHR-P individuals requires the administration of semistructured psychometric interviews by specialised preventive clinics. The Comprehensive Assessment of At-Risk Mental States (CAARMS/2006)^27^ is the original psychometric instrument that has been extensively validated to detect CHR-P individuals. The CAARMS/2006 has demonstrated excellent interrater reliability^28,29^ and very good prognostic accuracy (albeit at group-level) for transition to psychosis.^30^ However, its wider implementability in routine care is substantially constrained by resource-intensive assessment procedures that are available only in a few clinical research settings.^31^ Administering the CAARMS/2006 interview requires not only an extensive training for clinicians but also significant time—often averaging two hours—which poses logistical challenges in settings with limited resources.

To fill these gaps, this study, therefore, aimed to develop and validate the first short version of the CAARMS/2006, referred to as mini-CAARMS. By using advanced statistical methods we sought to develop and validate a mini-CAARMS that is psychometrically robust while retaining high accuracy against the full-length interview. We hope this study can provide a practical instrument to be used by many healthcare professionals worldwide to scale up preventive psychiatry in routine care, particularly in low-resource settings.

## Methods

### Design and Setting

This study leverages a cutting-edge dataset acquired in the UK and Italy as part of a Wellcome-Trust funded research programme, the E-Detection Tool for Emerging Mental Disorders (ENTER; detailed information about ENTER, including standardization and quality data procedures, can be found in a separate publication).^32^ The study received ethical approval from the East Midlands - Leicester Central Research Ethics Committee on 13^th^ January 2022 and the NHS Health Research Authority (NHRA) in the UK on March 24, 2022 (IRAS project ID: 298213) and by the Pavia Ethical Committee on 31^st^ August 2022 (protocol no. 0044135/22). All assessments for this study took place either at the Department of Psychosis Studies, Institute of Psychiatry, Psychology & Neuroscience, King’s College London; Institute of Neuroscience and Psychology, University of Glasgow, UK, or at the Department of Brain and Behavioral Sciences, University of Pavia, Italy.

### Participants

A total of 490 individuals (80% female sex; 73% white) undergoing CAARMS/2006 assessment and aged between 12 to 35 years (mean age 23.8±5) were recruited. We recruited from the general population, with all candidate participants first completing the 16-item Prodromal Questionnaire (PQ-16) pre-screening;^33^ which demonstrates excellent accuracy for detecting CHR-P in help-seeking samples (sensitivity and specificity ≈ 87%).^33^ We invited all individuals for the CAARMS/2006 interview who met the validated PQ-16 cut-off (≥ 6 endorsed items). To address potential effects of pre-screening, we also invited a randomly selected subset of individuals scoring below the PQ-16 cutoff (i.e., <6 endorsed items) for the CAARMS/2006 interview (n=97, 19.8% of the final sample). Participants below the PQ-16 cutoff did not differ by sex (p=.341) or ethnicity (p = .498). All participants provided written informed consent and received financial compensation according to the study protocol. Data were collected between 2022 and 2024.

### Study Procedures

All researchers administering the CAARMS/2006 were psychometrically trained in its use at each site, as part of the ENTER protocol.^32^ The research teams comprised psychologists, medical doctors, and postgraduate clinical research workers with experience in early psychosis assessment. Two senior clinical research academics supervised the study recruitment. The CAARMS/2006 was administered either virtually or in person over up to two separate visits.

### Measures

The gold-standard assessment instrument employed in this study to define a CHR-P status was the positive symptoms domain of the 2006 version *of the Comprehensive Assessment of At-Risk Mental States* (CAARMS/2006).^27^ The CAARMS/2006 includes only the positive symptoms that are used to determine inclusion groups: it assesses symptoms experienced across 60 different questions (i.e. 60 items) that are aggregated in to four overarching scales (each yielding a single score despite being made up of multiple questions): unusual thought content (13 items), non-bizarre ideas (11 items); perceptual abnormalities (22 items), and disorganized speech (14 items). The CAARMS/2006 requires aggregating multiple questions, covering multiple clinical subdomains into a single global rating of severity. Thereby each positive symptom scale is rated on a Likert scale from 0-6 for severity and an accompanying rating on a scale from 0-6 for frequency/duration. Additional ratings are made to assess the relationship of symptoms with substance use, distress (ranging from 0 to 100) and onset and offset of symptoms, however these do not contribute to the final psychometric intake.^27^ In this study, each of the 60 items was individually rated for severity, frequency, patterns, distress and onset/offset, so as well as the four core positive symptoms scales, we had independent ratings for all the individual symptoms that contributed to those four aggregate scores.

The four positive symptom scales are then used to detect those meeting an at-risk mental state (ARMS, which is an alternative acronym for CHR-P), and distinguish this from those not at risk for psychosis, or those who have already met the psychosis threshold. The ARMS group is subdivided into four risk groups:

(1) vulnerability group (i.e., presumed genetic vulnerability to psychotic disorder; GRD);
(2) attenuated psychotic symptoms – subthreshold intensity (APS_SI_);
(3) attenuated psychotic symptoms – subthreshold frequency (APS_SF_);
(4) brief limited intermittent psychotic symptoms (BLIPS) group.

Regarding the prognostic accuracy of the CAARMS/2006, the meta-analytical estimate of sensitivity was 0.86 (95% CI: 0.76–0.92) and specificity was 0.55 (95% CI: 0.48-0.63) at 2 years, with a 2-year AUC of 0.79 (95% CI: 0.75–0.83),^30^ indexing an adequate group-level prognostic accuracy.

In an effort to refine the instrument, a new version of the CAARMS/2006, the CAARMS 23^34^ was recently introduced. In the CAARMS 23, rather than aggregating them into the original 4 positive symptoms domains, there are 15 rated domains, likely further increasing administration time. Although the CAARMS 23 was not employed in the current study, its structure was considered in interpreting the main findings.

### Data Analysis

We followed the Transparent Reporting of a multivariable prediction model for Individual Prognosis Or Diagnosis (TRIPOD+AI) guidelines^35^ (Supplementary Table 1).

As a first step, CAARMS/2006 items (n=60) were dichotomized based on the established cutoff criteria to identify young people at risk of psychosis,^27^ resulting in a binary outcome of CHR-P status vs not at risk for psychosis. Six items with zero variance (i.e., all values equal to 0, indicating no risk) were removed: four items from the Disorganized Speech scale and one item each from the Non-bizarre Ideas and Perceptual Abnormalities scales (see Supplementary Table 2). To identify the optimal subset of items predictive of CAARMS/2006 outcomes, we employed advanced statistical methods based on regularized logistic regression with a least absolute shrinkage and selection operator (LASSO) penalty. LASSO requires determining a hyperparameter lambda, which controls the strength of regularization: larger values of lambda result in shrinkage of coefficients for a greater number of predictors considered irrelevant to zero, effectively selecting fewer predictors. We used the `glmnetˋ package (version 4.18).^36,37^

**Figure 1:**
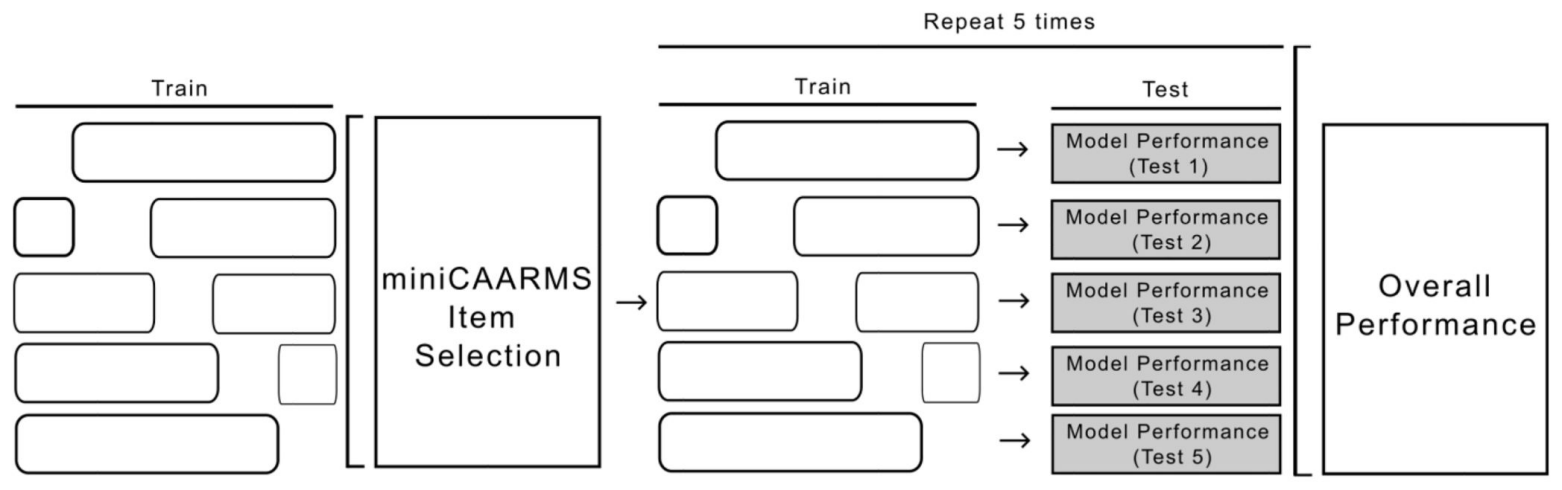
Modelling pipeline. First, data is split into five folds and five models are iteratively developed using four folds as training data. Items retained in all five of these models are selected to be the mini-CAARMS. Second, we train new models only including the items selected as part of the mini-CAARMS on the training data and test the performance on the fifth fold (test data). This second stage is repeated five times, with different participants being included in different folds on each repeat. Finally, the performance metrics across all five repeats across all five test folds are pooled to provide an estimate of overall performance.

We employed a modelling pipeline to select a consistent set of CAARMS/2006 items and assess detection performance, summarised below and in Figure 1.

#### Item selection

We used five-fold nested cross-validation to select a consistent subset of items representing the mini-CAARMS. The dataset was split into five cross-validation folds. Four folds was used to train a model, iteratively leaving one fold as test data. The hyperparameter lambda was optimised within the training data, selecting the minimum lambda value that maximized partial likelihood from a range of possible values. Once the optimal lambda was selected, the final model was retrained on the entire training dataset. This led to five models with five sets of retained items. We considered items to be “consistent” when they were retained by LASSO across four of the five cross-validation folds. This suggests that these items are useful for detection and stable, thus these items may generalize better to new clinical populations. This list of consistent selected items was considered the mini-CAARMS and used in the proceeding analytic steps.

#### Detection performance

We used repeated nested cross-validation to assess the ability of the mini-CAARMS to discriminate between CHR-P individuals vs those not at risk. As before, the dataset was split into five cross-validation folds. Lambda was optimised and a final model was fitted on the training data using the mini-CAARMS items only.

This model was then applied on the test data and performance metrics were calculated. Performance metrics computed for each fold contrasted the mini-CAARMS against the gold standard (i.e. the 60-items CAARMS/2006). These metrics included Cohen’s kappa (κ),^38^ Harrell’s C-index (concordance index),^39^ sensitivity, specificity, balanced accuracy, positive predictive value (PPV), negative predictive value (NPV), and F_1_ score (i.e., the harmonic mean of precision and recall).^39^ κ values were interpreted as follows: 0.01–0.20 indicating none-to-slight agreement, 0.21–0.40 fair agreement, 0.41–0.60 moderate agreement, 0.61–0.80 substantial agreement, and 0.81–1.00 almost perfect agreement.^38^ C-statistic values ≥ 0.70 indicate adequate ability to discriminate between risk profiles.^40^

This resulted in five sets of performance metrics as all cross-validation folds were used as test data. This process was repeated five times, with different dataset splits resulting in different participants being included in different folds each time. In total, 25 sets of performance metrics were generated. Mean and 95% CIs were calculated as a measure of overall model performance.

Within the CAARMS/2006 scoring system, individuals qualify for a CHR-P status if they meet the criteria on any one of the 60 items. The scoring system of the mini-CAARMS align with that of the full CAARMS: endorsing a single item retained in the mini-CAARMS is sufficient to classify an individual as CHR-P. Therefore, we used a threshold of 0 to assess classification performance. Consequently, this eliminates the possibility of false positives—thus yielding a specificity of 100%—but introduces the potential for false negatives if some individuals endorse only those items that have been removed by LASSO regularisation.

#### Prognostic performance

Finally, we simulated the 2-year prognostic accuracy of the mini-CAARMS for predicting psychosis onset. We conducted a Monte Carlo simulation using beta-distributed priors for sensitivity and specificity. Meta-analytic estimates of prognostic sensitivity (0.93) and specificity **(**0.58) for the full CAARMS/2006^30,41^ and diagnostic sensitivity and specificity of the mini-CAARMS compared to the full 60-items CAARMS/2006 were assumed to follow beta distributions, with parameters derived from reported confidence intervals. Where values were exactly 1, they were treated as a fixed value. For each of 10,000 simulations, we sampled from these distributions and computed the prognostic sensitivity and specificity:

$$\begin{array}{c} {\text{Se}}_{\text{prognostic}\;}={\text{Se}}_{\text{metaCAARMS}/2006}*{\text{Se}}_{\text{mini-CAARMS}\;}+\left(1-{\text{Sp}}_{\text{metaCAARMS}/2006}\right)*\left(1-{\text{Sp}}_{\text{mini-CAARMS}\;}\right)\\ {\text{Sp}}_{\text{prognostic}\;}={\text{Sp}}_{\text{metaCAARMS}/2006}*{\text{Sp}}_{\text{mini-CAARMS}\;}+\left(1-{\text{Se}}_{\text{metaCAARMS}/2006}\right)*\left(1-{\text{Se}}_{\text{mini-CAARMS}\;}\right) \end{array}$$

The posterior distributions of these estimates were then summarized using means and bootstrapped 95% CIs derived from 1,000 bootstraps with replacement (boot package version 1.3-31).

### Sensitivity analyses

First, we repeated the above analytic pipeline including a smaller selection of items that were present in all five cross-validation folds – representing the ultra-mini-CAARMS.

Second, to explore false negatives, the complete dataset, independently of LASSO’s variable selection, was used to identify individuals classified as ‘not at risk’ by either the mini-CAARMS or the ultra-mini-CAARMS despite fulfilling the full CAARMS/2006 risk criteria. The endorsement frequencies of the items omitted in these abbreviated versions were examined to determine whether any particular items were systematically endorsed by these misclassified cases.

Third, to assess algorithmic bias relating to ethnicity and sex, we re-estimated discrimination and classification metrics in (i) White and non-White participants; and (ii) male and female participants.

All analyses were performed in R (version 4.4.2; The R Foundation for Statistical Computing Platform, 2024). Sample size requirements for the model were calculated post hoc with the `psampsizeˋ (v.1.1.3) package.

## Results

### Sample characteristics

Of the 490 individuals interviewed with the CAARMS/2006, 120 were identified as meeting CHR-P status (APS_SI_, *n* = 106; BLIPS, *n* = 4; APS_SF_, *n* = 1, GRD only n=9), 358 were not at risk and 12 already met criteria for psychosis. The 9 individuals who met the GRD only criteria were not considered as at risk in our analyses because GRD status is determined by family history and functional decline rather than by the positive-symptom-based criteria used in this study. Sample size calculations indicated power was slightly lower than required for the main analyses (n=516, 124 events required).

### Mini-CAARMS

#### Item selection

Regularized logistic regression with a LASSO penalty identified 23 items (out of the 60 comprising the original CAARMS/2006) with the highest predictive value, which constitute the mini-CAARMS. Among these, 8 belong to the Non-Bizarre Ideas scale, 7 to the Unusual Thought Content scale, 7 to the Perceptual Abnormalities scale, and 1 to the Disorganized Speech scale. The full mini-CAARMS is provided in Supplementary Appendix 1 but a summary is presented in Table 1.

#### Detection Performance

The mini-CAARMS demonstrated excellent performance when compared to the full CAARMS/2006. The concurrent validity of the mini-CAARMS was high, with Cohen’s κ equal to 0.90 (95% CI: 0.86–0.95), indicating almost perfect agreement. Harrell’s C index was 0.93 (95% CI: 0.90–0.96), indicating excellent discriminative ability. The sensitivity was 95.6% (95% CI: 93.8–97.4), specificity was 100%, balanced accuracy was 97.8% (95% CI: 96.9–98.7), PPV was 100%, NPV was 86.3% (95% CI: 80.5–92.1), and the F_1_ score was 97.7% (95% CI: 96.8–98.7).

There were four false negatives. Misclassification analysis identified six omitted items; each was endorsed exactly once across these four false negatives, meaning each item was endorsed by 25% of this subgroup (see Supplementary Table 3 for item content). Individuals classified as No Risk had a mean severity of 10.1 (SD = 9.1), whereas those classified as Risk had a mean severity of 31.7 (SD = 21.2); *t*_(137)_ = –11, *p* < .001. The 95% confidence interval for the mean difference was between –25.5 and –17.7. These results indicate not only a statistically significant but also a clinically meaningful difference in symptom severity between the two groups.

#### Prognostic Performance

The Monte Carlo simulation produced similar estimates for the prognostic accuracy of the mini-CAARMS to the meta-analytic estimates for the full CAARMS/2006. Prognostic sensitivity was 89.0% (95% CI: 88.6-89.4) and specificity was 57.9% (95% CI: 57.5-58.3)

### Sensitivity analysis

#### Ultra-mini-CAARMS

Sample size calculations indicated sensitivity analyses were well-powered (n=281, 68 events required).

##### Item selection

Twelve items from the mini-CAARMS were consistently retained in all five cross-validation folds and constituted the ultra-mini-CAARMS. Of these, 5 items belonged to the Perceptual Abnormalities scale, 4 to the Non-Bizarre Ideas scale, and 3 to the Unusual Thought Content scale. The full ultra-mini-CAARMS is provided in Supplementary Appendix 2, but a summary is presented in Table 1.

##### Detection Performance

The ultra-mini-CAARMS demonstrated good performance when compared to the full CAARMS/2006. Cohen’s κ was 0.90 (95% CI: 0.86–0.95), indicating substantial agreement. Harrell’s C index was 0.91 (95% CI: 0.88–0.94), indicating excellent discriminative ability. The sensitivity was 94.4% (95% CI: 92.3–96.5), specificity was 100%, balanced accuracy was 97.2% (95% CI: 96.2–98.2), PPV was 100%, NPV was 82.2% (95% CI: 75.6–88.9), and the F_1_ score was 97.7% (95% CI: 96.8–98.7).

##### Prognostic performance

The Monte Carlo simulation estimated that the prognostic accuracy of the ultra-mini-CAARMS is comparable to that of the full CAARMS/2006. Prognostic sensitivity was 87.8% (95% CI: 87.3-88.2) and specificity was 57.9% (95% CI: 57.5-58.3).

##### Misclassification analysis

Misclassification analysis revealed an additional 16 false negatives with the ultra-mini-CAARMS beyond the 4 observed with the mini-CAARMS (total = 20). Among the false negatives, examination of omitted CAARMS/2006 items showed that eight items were each endorsed by two individuals (10% of false negatives), while a further 17 items were endorsed by one individual (5% of false negatives). No single item overwhelmingly contributed to misclassification.

##### Algorithmic bias assessment

Discrimination and classification metrics were computed separately for participants who identified as White and Non-White. The mini-CAARMS achieved excellent and largely comparable performance in White and non-White participants. Sensitivity was 97.8% in White participants and 93.1% in non-White participants; balanced accuracy and C-index remained ≥ 0.96 in both groups. Thus, there was no evidence of systematic misclassification by ethnicity. When the same metrics were computed for female and male participants, sensitivity, F_1_, balanced accuracy and C-index again remained in the excellent range (all ≥ 0.96), with no material difference between sexes. These findings confirm that the mini-CAARMS maintains high classification performance without detectable sex biases. See supplementary Table 5.

## Discussion

This study developed and validated the first abbreviated version of the CAARMS/2006 interview (mini-CAARMS) that maintains high detection accuracy for identifying CHR-P individuals while substantially reducing administration time. The mini-CAARMS demonstrates strong concordance with the original CAARMS/2006, suggesting minimal compromise in accurately identifying at-risk states. These findings have significant implications for clinical practice and research, particularly in settings where time, personnel, or financial constraints pose challenges to routinely administering comprehensive assessments.

The mini-CAARMS (along with the additional version of the ultra-mini-CAARMS; see the Supplementary Material for copies of each scale and scoring instructions) showed very high concordance with the original CAARMS/2006, indicating that even when substantially fewer questions are asked, accurate identification of at-risk states is preserved. This finding was somewhat expected based on the structural architecture of the CAARMS/2006, which is characterised by aggregate items for a given domain. Both mini- and ultra-mini versions achieved strong sensitivity. Notably, the mini-CAARMS, which retains fewer than half of the original items, demonstrated almost perfect agreement with the full CAARMS/2006, suggesting that its retained items capture the most critical clinical features of risk status. Although the ultra-mini-CAARMS requires a more pronounced reduction in items, it maintains a high level of diagnostic accuracy, indicating an acceptable balance between efficiency and precision. By ensuring consistency of items, using repeated nested cross-validation and following gold standard methodological guidelines, these results are more likely to generalise to other similar samples.

Importantly, both abbreviated versions show comparable prognostic accuracy to the full CAARMS/2006 in our simulated analyses. Specifically, the meta-analytical estimates of CAARMS sensitivity and specificity at 2 years were 93% (95% CI: 87%–96%) and 58% (95% CI: 50%–66%), respectively, whereas sensitivity and specificity based on our data for both abbreviated versions were >87% and 57%, respectively. These results suggest that both the mini-CAARMS and ultra-mini-CAARMS retain a largely similar prognostic accuracy to the full CAARMS/2006 in detecting future transitions to psychosis. However, our misclassification analysis indicates that the ultra-mini-CAARMS misses more cases (i.e., yields additional false negatives) than the mini-CAARMS. Consequently, while the ultra-mini-CAARMS may still offer a viable option in contexts with severely limited resources, the mini-CAARMS should be preferred in standard clinical settings to minimize the risk of misclassification. Importantly, neither abbreviated interview showed evidence of systematic demographic (i.e., sex or ethnic) biases.

The mini- and ultra-mini-CAARMS were designed to provide clinicians and researchers with time-efficient yet valid alternatives to the original CAARMS/2006. Indeed our analyses preserve the structure, administration, and scoring procedures of the original interview, so no additional training beyond that required for the full CAARMS/2006 is necessary. While a dedicated, and streamlined training focusing on the 23 items of the mini-CAARMS could be considered, we recommend that it should be preceded (or at least complemented) by a full CAARMS/2006 training to provide broader psychopathological knowledge and psychometric expertise. We also acknowledge that the shorter format can limit opportunity to build personal rapport and to validate the young person’s unusual experiences. For this reason, we would emphasise the importance of considering a standard psychiatric interview before administering the mini-CAARMS and acknowledge and validate any distress or confusion the person may be experiencing in relation to their symptoms.

Beyond the CAARMS framework, a few initiatives have sought to streamline psychosis-risk interviews, and it is useful to position the mini-CAARMS against these existing tools. The Mini-SIPS, a short clinical version of the Structured Interview for Psychosis-risk Syndromes, reduces administration and training to roughly 30–60 minutes and a brief on-line module, respectively.^42^ However, to the best of our knowledge, no prospective validation of diagnostic or prognostic performance of the MINI-SIPS has yet been published. In contrast, the recently developed Positive SYmptoms and Diagnostic Criteria for the CAARMS Harmonized with the SIPS (PSYCHS),^43^ a single 15-item battery that fully aligns CAARMS and SIPS psychosis-conversion criteria, still requires 60–90 minutes and has not been empirically benchmarked against either parent interview. Compared with these approaches, the 23-item mini-CAARMS preserves the original CAARMS scoring logic, demonstrates near-perfect diagnostic agreement (κ=.90) with the full interview, and can be delivered in ~35 minutes. Future head-to-head studies could clarify whether the additional content retained in the mini-CAARMS translates into superior sensitivity or prognostic value relative to the leaner Mini-SIPS and to the broader, harmonised PSYCHS, although we envisage that these instruments could and should be used complementarily and not as competing alternatives.

Intervening at earlier stages of psychosis, particularly before its onset, is critical for modifying the illness trajectory.^44,45^ Within preventive psychiatry, primary indicated prevention on CHR-P individuals aims to avert or delay full-blown psychosis.^46^ While prognostic prediction is well established in other medical domains through biomarkers, currently no biological tests exist for psychosis.^47^ Therefore psychometric interviews, such as the mini-CAARMS, are the essence of preventive assessment in routine care today. By substantially reducing the interview duration without compromising sensitivity or specificity, the mini-CAARMS and ultra-mini-CAARMS enable broader adoption of CHR-P assessments in diverse clinical settings, including non-specialist community mental health centres and primary care. This is particularly pertinent given the escalating pressures on healthcare systems in the UK and across Europe, which face tighter budgets and a growing shortfall of qualified staff.^48,49^ Lengthy clinical interviews can exacerbate these financial and logistical strains, slowing down detection and referral processes. Even in the UK—where preventive infrastructures are comparatively well developed—a recent clinical audit found that most CHR-P services manage very low caseloads partly because the full CAARMS/2006 is so time-intensive.^10^ By offering a briefer yet accurate assessment, the mini-CAARMS may help expand CHR-P detection capacity while alleviating service bottlenecks, ultimately promoting earlier and more cost-effective clinical interventions.

From a research standpoint, shorter interviews could facilitate screening of larger populations and therefore increase sample sizes while at the same time reducing participant burden in prospective studies, this enhancing retention and data quality over time. Large-scale studies investigating novel biomarkers, risk factors, or interventions for CHR-P populations stand to benefit from streamlined assessments that maintain robust psychometric properties.^50^ Moreover, the mini- and ultra-mini-CAARMS can be more easily integrated into telehealth assessments to broaden their reach and further expedite participant screening, particularly in epidemiological or community-based research. This is promising not only for high-resource settings but also for low- and middle-resource settings where telemedicine infrastructure is being expanded.^51^

Finally, our LASSO-based approach, which automatically selects variables of greatest predictive importance, has provided information about which CAARMS/2006 items are most predictive of CHR-P status, thereby offering preliminary insights into the relevance of particular subdomains. Notably, speech-related items were almost entirely absent from our final models, suggesting that disorganized speech, at least as measured by the CAARMS/2006, may be insufficiently specific for identifying CHR-P individuals. Recognizing that some subdomains might be less central to detecting CHR-P provides a useful starting point for further refining the CHR-P and CAARMS frameworks. Future research, including dedicated factor analyses and external validation across varied clinical populations, is warranted to validate these initial indications and potentially streamline risk detection for psychosis even further.

This study has limitations. Firstly, the mini-CAARMS performance was examined only on a dichotomized outcome (CHR-P vs. not at risk), rather than across CHR-P subgroups (i.e., attenuated psychotic symptoms subthreshold intensity, attenuated psychotic symptoms subthreshold frequency, brief limited intermittent psychotic symptoms, and psychosis threshold). Nevertheless, from a clinical standpoint, the CAARMS/2006 is primarily intended to determine whether an individual meets the criteria for a CHR-P, which is the primary gateway to preventive care.

Secondly, while overall performance is high with all individuals meeting ultra-mini-CAARMS and mini-CAARMS criteria also meeting the full CAARMS/2006 criteria, it is not possible to distinguish between false negatives and true negatives without administering the full CAARMS/2006 assessment. However, our analysis of omitted items showed that the individuals who were misclassified each endorsed different items from the pool that were omitted. There was no single question that, if reinstated, would have prevented all false negatives. Instead, the misclassifications were caused by a mix of less-performing questions that—collectively, rather than individually—contributed to missing a small number of at-risk cases.

Thirdly, we do not currently have follow-up data for this cohort and as such we are unable to compare prognostic accuracy of the ultra-mini-CAARMS and mini-CAARMS against the full CAARMS. However, we have addressed this by producing simulated estimates of prognostic accuracy using the state-of-the-art diagnostic accuracy meta-analytic estimates based on the most significant prospective CHR-P cohorts worldwide (892 CHR-P individuals vs. 984 individuals not at risk, 2 years follow-up).

Fourthly, our community sample was heavily skewed toward female participants (80%) and White participants (73%). Although sex and ethnicity did not significantly affect the results, there was a limited statistical power to detect subtle performance differences. At the same time, it is worth noting that recruitment took place across three independent UK/Italian sites (i.e., Glasgow, London, and Pavia) rather than a single center, providing geographic and health-system diversity and additional support for the generalizability of our findings.

Fifthly, participants were enrolled from the community population through a two-stage pre-screening procedure in which the PQ-16 served as the initial screen. Although we did include a random subsample of individuals not meeting the PQ-16 cutoff, future replication studies are needed to confirm the psychometric properties of the mini-CAARMS in cohorts that are not pre-screened. We employed a community population because we primarily aimed at developing new assessment instruments that can expand the detection of those at risk in the larger scale (i.e. outside clinical samples).^52,53^ Future replications in help-seeking clinical samples will test the psychometric properties of the abbreviated CAARMS versions. However, pre-screening with PQ-16 significantly improves sensitivity of CHR-P instruments compared to studies in samples without pre-screening.^41^ As such, we do not expect substantially lower accuracy if the ultra-mini- and mini-CAARMS are restricted to clinical samples (vice versa, we would have expected lower accuracy when applied to community samples had the ultra-mini- and mini-CAARMS been developed using highly risk enriched clinical samples only).

Future research should also better investigate the mini-CAARMS in comparison to the CAARMS 23. However, our findings provide a useful benchmark. Although the mini- and ultra-mini-CAARMS were developed using the original CAARMS/2006, all the items they retain were also incorporated into the CAARMS 23 (with varying degrees of rewording; see Supplementary Table 4). This observation indirectly confirms that the CAARMS 23 has preserved the most valuable items, albeit its factorial structure has not been empirically tested. At the same time, some questions identified by our analyses as less efficient (e.g., “Is anyone in love with you?”, “Have you ever had the feeling that something odd is going on or that something is wrong?”) still remain in the CAARMS 23.

Finally, future research should formally assess how these abbreviated versions perform in diverse clinical and community settings, examining factors such as administration time, user acceptability, and integration into established early intervention pathways. Additionally, exploring cost-effectiveness and the potential impact on service efficiency, such as increased screening capacity or quicker referrals, would further clarify the practical benefits of these new tools.

## Conclusions

The mini-CAARMS substantially reduces the administration burden while preserving its accuracy in identifying CHR-P individuals. The mini-CAARMS is recommended for most clinical and research applications. This short version holds promise for scaling up the early detection of CHR-P states, particularly in settings with limited resources, thereby facilitating timely preventive intervention and potentially improving clinical outcomes.

## Supplementary Material

Supplementary Materials

## Figures and Tables

**Table 1:** Questions included in the mini-CAARMS and ultra-mini-CAARMS

	mini-CAARMS	ultra-mini CAARMS
	*Unusual thought content*	
1	Have you had the feeling that something odd is going on that you can’t explain? What is it like?	**✓**
2	Do familiar surroundings feel strange?	
3	Do you feel that others, or the world, have changed in some way?	
4	Have you felt that things that were happening around you had a special meaning, or that people were trying to give you a message? IF YES: What is it like? How did it start?	
5	Have you felt that someone, or something, outside yourself has been controlling your thoughts, feelings, actions or urges?	**✓**
6	Do you get any strange sensations in your body?	
7	Can other people read your mind?	**✓**
	*Non-bizarre ideas*	
8	Has anybody been giving you a hard time or trying to hurt you? IF YES: How do you know this?	**✓**
9	Do you feel like people have been talking about you, laughing at you, or watching you? IF YES: How do you know this?	**✓**
10	Have you had the feeling that something odd is going on with your body that you can’t explain? What is it like?	**✓**
11	Do you feel that your body has changed in some way, or that there is a problem with your body shape?	
12	Do you feel you deserve punishment for anything you have done wrong?	**✓**
13	Have you ever felt that you, or a part of you, did not exist, or was dead?	
14	Do you ever feel that the world does not exist?	
15	Are you a jealous person? Do you worry about relationships that your spouse/girlfriend/boyfriend has with other people?	
	*Perceptual abnormalities*	
16	Is there a change in the way things look to you?	**✓**
17	Do you have visions, or see things that may not really be there? IF YES: What do you see? At the time that you see these things, how real do they seem? Do you realise that they are not real at the time, or only later?	**✓**
18	Do you ever hear things that other people seem not to (such as sounds or voices)? IF YES: What do you hear? At the time that you hear these things, how real do they seem? Do you realise that they are not real at the time, or only later?	**✓**
19	Does your sense of smell seem to be different, such as more, or less intense, than usual?	
20	Do you ever smell things that other people don’t notice? At the time, do these smells seem real? Do you realise they are not real at the time, or only later?	**✓**
21	Do you ever get strange feelings on, or just beneath, your skin? IF YES: At the time that you feel these things, how real do they seem? Do you realise they are not real at the time, or only later?	**✓**
22	Do you feel/think that there is a problem with some part, or all of your body, i.e. that it looks different to others, or is different in some way? IF YES: How real does this seem?	
	*Disorganised speech*	
23	Do you have trouble finding the correct word at the appropriate time?	
