# Supplementary material for: The Mini-CAARMS: Development and Validation of a Short Version of the Comprehensive Assessment of AT Risk Mental States to Facilitate Preventive Psychiatry
Source: Schizophr Bull. Author manuscript; Available in PMC 2026 Mar 27. (PMC7618934; doi:10.1093/schbul/sbaf146)
Supplement: Supplementary Materials [file EMS213004-supplement-Supplementary_Materials.docx]

**Supplementary Table 1.** TRIPOD + AI Guidelines

| **Section/Topic** | | | **Item** | | **Development/Evaluation** | **Checklist Item** | **Reported on page** |
| --- | --- | --- | --- | --- | --- | --- | --- |
| **TITLE** | | | | | | |  |
| *Title* | 1 | D;E | | Identify the study as developing or evaluating the performance of a multivariable prediction model, the target population, and the outcome to be predicted | | | 1 |
| **ABSTRACT** | | | | | | | |
| *Abstract* | 2 | D;E | | See TRIPOD+AI for Abstracts checklist | | | 2 |
| **INTRODUCTION** | | | | | | | |
| *Background* | 3a | D;E | | Explain the healthcare context (including whether diagnostic or prognostic) and rationale for developing or evaluating the prediction model, including references to existing models | | | 3 |
|  | 3b | D;E | | Describe the target population and the intended purpose of the prediction model in the context of the care pathway, including its intended users (e.g., healthcare professionals, patients, public) | | | 3 |
|  | 3c | D;E | | Describe any known health inequalities between sociodemographic groups | | | 16 |
| *Objectives* | 4 | D;E | | Specify the study objectives, including whether the study describes the development or validation of a prediction model (or both) | | | 4 |
| **METHODS** | | | | | | | |
| *Data* | 5a | D;E | | Describe the sources of data separately for the development and evaluation datasets (e.g., randomised trial, cohort, routine care or registry data), the rationale for using these data, and representativeness of the data | | | 5 |
|  | 5b | D;E | | Specify the dates of the collected participant data, including start and end of participant accrual; and, if applicable, end of follow-up | | | 5 |
| *Participants* | 6a | D;E | | Specify key elements of the study setting (e.g., primary care, secondary care, general population) including the number and location of centres | | | 5 |
|  | 6b | D;E | | Describe the eligibility criteria for study participants | | | 5 |
|  | 6c | D;E | | Give details of any treatments received, and how they were handled during model development or evaluation, if relevant | | | 5-6 |
| *Data preparation* | 7 | D;E | | Describe any data pre-processing and quality checking, including whether this was similar across relevant sociodemographic groups | | | 7 |
| *Outcome* | 8a | D;E | | Clearly define the outcome that is being predicted and the time horizon, including how and when assessed, the rationale for choosing this outcome, and whether the method of outcome assessment is consistent across sociodemographic groups | | | 5-6 |
|  | 8b | D;E | | If outcome assessment requires subjective interpretation, describe the qualifications and demographic characteristics of the outcome assessors | | | 5-6 |
|  | 8c | D;E | | Report any actions to blind assessment of the outcome to be predicted | | | n/a |
| *Predictors* | 9a | D | | Describe the choice of initial predictors (e.g., literature, previous models, all available predictors) and any pre-selection of predictors before model building | | | 6 |
|  | 9b | D;E | | Clearly define all predictors, including how and when they were measured (and any actions to blind assessment of predictors for the outcome and other predictors) | | | 6 |
|  | 9c | D;E | | If predictor measurement requires subjective interpretation, describe the qualifications and demographic characteristics of the predictor assessors | | | n/a |
| *Sample size* | 10 | D;E | | Explain how the study size was arrived at (separately for development and evaluation), and justify that the study size was sufficient to answer the research question. Include details of any sample size calculation | | | 11 |
| *Missing data* | 11 | D;E | | Describe how missing data were handled. Provide reasons for omitting any data | | | 7 |
| *Analytical methods* | 12a | D | | Describe how the data were used (e.g., for development and evaluation of model performance) in the analysis, including whether the data were partitioned, considering any sample size requirements | | | 7-8 |
|  | 12b | D | | Depending on the type of model, describe how predictors were handled in the analyses (functional form, rescaling, transformation, or any standardisation). | | | 8 |
|  | 12c | D | | Specify the type of model, rationale^2^, all model-building steps, including any hyperparameter tuning, and method for internal validation | | | 8 |
|  | 12d | D;E | | Describe if and how any heterogeneity in estimates of model parameter values and model performance was handled and quantified across clusters (e.g., hospitals, countries). See TRIPOD-Cluster for additional considerations^3^ | | | 8 |
|  | 12e | D;E | | Specify all measures and plots used (and their rationale) to evaluate model performance (e.g., discrimination, calibration, clinical utility) and, if relevant, to compare multiple models | | | 8-9 |
|  | 12f | E | | Describe any model updating (e.g., recalibration) arising from the model evaluation, either overall or for particular sociodemographic groups or settings | | | n/a |
|  | 12g | E | | For model evaluation, describe how the model predictions were calculated (e.g., formula, code, object, application programming interface) | | | 9-10 |
| *Class imbalance* | 13 | D;E | | If class imbalance methods were used, state why and how this was done, and any subsequent methods to recalibrate the model or the model predictions | | | n/a |
| *Fairness* | 14 | D;E | | Describe any approaches that were used to address model fairness and their rationale | | | 9 |
| *Model output* | 15 | D | | Specify the output of the prediction model (e.g., probabilities, classification). Provide details an rationale for any classification and how the thresholds were identified | | | 8-10 |
| *Training versus*  *evaluation* | 16 | D;E | | Identify any differences between the development and evaluation data in healthcare setting, eligibility criteria, outcome, and predictors | | | 8 |
| *Ethical approval* | 17 | D;E | | Name the institutional research board or ethics committee that approved the study and describe the participant-informed consent or the ethics committee waiver of informed consent | | | 10 |
| **OPEN SCIENCE** | | | | | | | |
| *Funding* | 18a | D;E | | Give the source of funding and the role of the funders for the present study | | | 23 |
| *Conflicts of interest* | 18b | D;E | | Declare any conflicts of interest and financial disclosures for all authors | | | 24 |
| *Protocol* | 18c | D;E | | Indicate where the study protocol can be accessed or state that a protocol was not prepared | | | 7 |
| *Registration* | 18d | D;E | | Provide registration information for the study, including register name and registration number, or state that the study was not registered | | | 7 |
| *Data sharing* | 18e | D;E | | Provide details of the availability of the study data | | | 23 |
| *Code sharing* | 18f | D;E | | Provide details of the availability of the analytical code^4^ | | | 23 |
| **PATIENT & PUBLIC INVOLVEMENT** | | | | | | | |
| *Patient & Public Involvement* | 19 | D;E | | Provide details of any patient and public involvement during the design, conduct, reporting, interpretation, or dissemination of the study or state no involvement. | | | n/a |
| **RESULTS** | | | | | | | |
| *Participants* | 20a | D;E | | Describe the flow of participants through the study, including the number of participants with and without the outcome and, if applicable, a summary of the follow-up time. A diagram may be helpful. | | | 11 |
|  | 20b | D;E | | Report the characteristics overall and, where applicable, for each data source or setting, including the key dates, key predictors (including demographics), treatments received, sample size, number of outcome events, follow-up time, and amount of missing data. A table may be helpful. Report any differences across key demographic groups. | | | 11 |
|  | 20c | E | | For model evaluation, show a comparison with the development data of the distribution of important predictors (demographics, predictors, and outcome). | | | 11 |
| *Model development* | 21 | D;E | | Specify the number of participants and outcome events in each analysis (e.g., for model development, hyperparameter tuning, model evaluation) | | | 11, 19-20 |
| *Model specification* | 22 | D | | Provide details of the full prediction model (e.g., formula, code, object, application programming interface) to allow predictions in new individuals and to enable third-party evaluation and implementation, including any restrictions to access or re-use (e.g., freely available, proprietary)^5^ | | | Supplementary |
| *Model performance* | 23a | D;E | | Report model performance estimates with confidence intervals, including for any key subgroups (e.g., sociodemographic). Consider plots to aid presentation. | | | 21-22 |
|  | 23b | D;E | | If examined, report results of any heterogeneity in model performance across clusters. See TRIPOD Cluster for additional details^3^. | | | 21-22 |
| *Model updating* | 24 | E | | Report the results from any model updating, including the updated model and subsequent performance | | | n/a |
| **DISCUSSION** | | | | | | | |
| *Interpretation* | 25 | D;E | | Give an overall interpretation of the main results, including issues of fairness in the context of the  objectives and previous studies | | | 14 |
| *Limitations* | 26 | D;E | | Discuss any limitations of the study (such as a non-representative sample, sample size, overfitting, missing data) and their effects on any biases, statistical uncertainty, and generalizability | | | 16 |
| *Usability of the model in the context of current care* | 27a | D | | Describe how poor quality or unavailable input data (e.g., predictor values) should be assessed and handled when implementing the prediction model | | | 7 |
|  | 27b | D | | Specify whether users will be required to interact in the handling of the input data or use of the model, and what level of expertise is required of users | | | 7 |
|  | 27c | D;E | | Discuss any next steps for future research, with a specific view to applicability and generalizability of the model | | | 15 |

**Supplementary Table 2.** Items with zero variance (i.e., all values equal 0, indicating no risk)

|  | **Item** |
| --- | --- |
| N | Is anyone in love with you? Who? How do you know this? Do you return his/her feelings? |
| P | Have you noticed any change in your bodily sensations, such as increased, or reduced intensity? |
| S | Do you ever find yourself repeating the words of others? |
| S | Do they go off the subject often and get lost in their words? |
| S | Do they appear to have difficulty finding the right words? |
| S | Do they repeat words that you have used or adopt strange words (or 'non-words') in the course of regular conversations? |

**Supplementary Table 3.** Content of CAARMS Items Omitted by the mini‐CAARMS That Contributed to Meeting Full CAARMS Criteria

|  | **Item content** |
| --- | --- |
| U | Do you get any strange sensations in your body? IF YES: Do you know what causes them? Could it be due to other people or forces outside yourself? |
| P | Are there alterations in colour, or brightness of objects (things seeming brighter, or duller in colour)? |
| P | Are there alterations in the size and shape of objects? |
| P | Do you ever seen things that others can't, or don't seem to? IF YES: What do you see? At the time that you see these things, how real do they seem? Do you realise that they are not real at the time, or only later? |
| P | Does your sense of smell seem to be different, such as more, or less intense, than usual? |
| P | Do you ever get any odd taste in your mouth? IF YES: At the time that you taste these things, how real do they seem? Do you realise that they are not real at the time, or only later? |

**Supplementary Table 4.** Comparison between mini- and ultra-mini-CAARMS Item Contents and CAARMS 23 Respective Items

|  |  | **CAARMS/2006** |  | |  |  | **CAARMS 23** | |  |
| --- | --- | --- | --- | --- | --- | --- | --- | --- | --- |
|  | **mini-CAARMS** | | | **ultra-mini**  **CAARMS** | |  |  |  | |
| U | Have you had the feeling that something odd is going on that you can’t explain? What is it like? | | | ✔ | |  | U | Have you ever had the feeling that something odd is going on or that something is wrong? | |
| U | Do familiar surroundings feel strange? | | |  | |  | U | Do familiar people or surroundings ever seem strange? | |
| U | Do you feel that others, or the world, have changed in some way? | | |  | |  | U | Do you feel that you,^1^ others, or the world have changed in some way? | |
| U | Have you felt that things that were happening around you had a special meaning, or that people were trying to give you a message? IF YES: What is it like? How did it start? | | |  | |  | U | Have you felt that things that were happening around you had a special meaning just for you? | |
| U | Have you felt that someone, or something, outside yourself has been controlling your thoughts, feelings, actions or urges? | | | ✔ | |  | U | Have you ever felt that some person or force outside yourself has been controlling or interfering with your thoughts, feelings, actions or urges? | |
| U | Do you get any strange sensations in your body? | | |  | |  | P | Do you ever get strange feelings in your body? | |
| U | Can other people read your mind? | | | ✔ | |  | U | Do you ever think that people might be able to read your mind? Or that you could read other people’s minds? | |
| N | Has anybody been giving you a hard time or trying to hurt you? IF YES: How do you know this? | | | ✔ | |  | U | Has anybody been giving you a hard time or trying to hurt you? Do you have a sense of who that might be? | |
| N | Do you feel like people have been talking about you, laughing at you, or watching you? IF YES: How do you know this? | | | ✔ | |  | U | Do you ever feel like people have been talking about you, laughing at you or thinking about you in a negative way? | |
| N | Have you had the feeling that something odd is going on with your body that you can’t explain? What is it like? | | | ✔ | |  | U | Have you had the feeling that something odd is going on with your body that you can’t explain? | |
| N | Do you feel that your body has changed in some way, or that there is a problem with your body shape? | | |  | |  | P | Do you ever feel that parts of your body have changed in some way, or that things are working differently? | |
| N | Do you feel you deserve punishment for anything you have done wrong? | | | ✔ | |  | G | Do you believe that you deserve to be punished in some way? | |
| N | Have you ever felt that you, or a part of you, did not exist, or was dead? | | |  | |  | U | Have you ever felt that you might not actually exist? Or that the world might not exist? | |
| N | Do you ever feel that the world does not exist? | | |  | |  |  |  |  |
| N | Are you a jealous person? Do you worry about relationships that your spouse/girlfriend/boyfriend has with other people? | | |  | |  | G | Has there ever been anyone in your life that you’ve been jealous of, for example a work colleague, friend or partner? What was it about these people that made you jealous? | |
|  |  |  |  |  |  |  | G | Did these people/your partner have any relationships with anyone that you worried about? | |
| P | Is there a change in the way things look to you? | | | ✔ | |  | P | Is there any change in the way things sound to you? | |
| P | Do you have visions, or see things that may not really be there? IF YES: What do you see? At the time that you see these things, how real do they seem? Do you realise that they are not real at the time, or only later? | | | ✔ | |  | P | Do you seem to feel more sensitive to light or do things that you see appear different in colour, brightness or dullness; or have they changed in some other way? Are there alterations in the size and shape of objects? Do they seem to be moving? | |
|  |  |  |  |  |  |  | P | Have you ever seen unusual things like flashes, flames, vague figures, shadows, or movement out of the corner of your eye? | |
|  |  |  |  |  |  |  | P | Do you ever “mis-see” things? | |
| P | Do you ever hear things that other people seem not to (such as sounds or voices)? IF YES: What do you hear? At the time that you hear these things, how real do they seem? Do you realise that they are not real at the time, or only later? | | | ✔ | |  | P | Do you ever hear unusual sounds like banging, clicking, hissing, clapping, ringing in your ears? | |
|  |  |  |  |  |  |  | P | Do you ever hear things that may not really be there? | |
|  |  |  |  |  |  |  | P | Do you ever hear a voice that others don’t seem to or can’t hear? Does it sound clearly like a voice speaking to you as I am now? Could it be your own thoughts or is it clearly a voice speaking out loud? | |
| P | Does your sense of smell seem to be different, such as more, or less intense, than usual? | | |  | |  | P | Does your sense of smell seem to be different, such as more, or less intense, than usual? | |
| P | Do you ever smell things that other people don’t notice? At the time, do these smells seem real? Do you realise they are not real at the time, or only later? | | | ✔ | |  | P | Do you ever smell things that other people don’t notice? At the time that you smell these things, how real do they seem? | |
| P | Do you ever get strange feelings on, or just beneath, your skin? IF YES: At the time that you feel these things, how real do they seem? Do you realise they are not real at the time, or only later? | | | ✔ | |  | P | Do you ever get strange feelings on, or just beneath, your skin? At the time that you feel these things, how real do they seem? | |
| P | Do you feel/think that there is a problem with some part, or all of your body, i.e. that it looks different to others, or is different in some way? IF YES: How real does this seem? | | |  | |  | P | Do you feel or think that there is a problem with some part, or all of your body? | |
|  |  |  |  |  |  |  | P | Do you feel or think that it looks different to others, or is different in some way? How real does it seem? | |
| S | Do you have trouble finding the correct word at the appropriate time? | | |  | |  | S | Do you have trouble finding the correct word at the appropriate time? | |

*Note.* G = ideas of guilt subscale; N = non-bizarre ideas subscale; P = perceptual abnormalities subscale; S = disorganised speech subscale for the mini-CAARMS and disorganised communication expression subscale for the CAARMS 23; U = unusual thought content scale for the mini-CAARMS and unusual thoughts and experiences subscale for the CAARMS 23.

^1^ The CAARMS item “Do you feel that you have changed in some way?” was not selected in the abbreviated version.

**Supplementary Table 5.** Classification Metrics of the mini-CAARMS Model Across Ethnicity and Sex Subgroups.

| Subgroup | n | TP | FN | FP | TN | Sensitivity | Specificity | PPV | NPV | F1 | Accuracy | BalancedAcc | AUC |
| --- | --- | --- | --- | --- | --- | --- | --- | --- | --- | --- | --- | --- | --- |
| Ethnicity |  |  |  |  |  |  |  |  |  |  |  |  |  |
| Non-White | 133 | 27 | 2 | 0 | 104 | 0.931 | 1.0 | 1.0 | 0.981 | 0.964 | 0.985 | 0.966 | 0.919–1 |
| White | 356 | 91 | 2 | 0 | 263 | 0.978 | 1.0 | 1.0 | 0.992 | 0.989 | 0.994 | 0.989 | 0.974–1 |
| Sex |  |  |  |  |  |  |  |  |  |  |  |  |  |
| Male | 97 | 14 | 0 | 0 | 83 | 1.0 | 1.0 | 1.0 | 1.0 | 1.0 | 1.0 | 1.0 | 1–1 |
| Female | 392 | 104 | 4 | 0 | 284 | 0.963 | 1.0 | 1.0 | 0.986 | 0.981 | 0.99 | 0.981 | 0.964–0.999 |
